# X-Linked Emery–Dreifuss Muscular Dystrophy: Study Of X-Chromosome Inactivation and Its Relation with Clinical Phenotypes in Female Carriers

**DOI:** 10.3390/genes10110919

**Published:** 2019-11-11

**Authors:** Emanuela Viggiano, Agnieszka Madej-Pilarczyk, Nicola Carboni, Esther Picillo, Manuela Ergoli, Stefania del Gaudio, Michal Marchel, Gerardo Nigro, Alberto Palladino, Luisa Politano

**Affiliations:** 1 Cardiomyology and Medical Genetics, Department of Experimental Medicine, University of Campania, 80138 Naples, Italy; 2 Neuromuscular Unit, Mossakowski Medical Research Centre, Polish Academy of Sciences, 00-901 Warsaw, Poland; 3 Neurology Department, Hospital San Francesco of Nuoro, 08100 Nuoro, Italy; 4 Department of Experimental Medicine, University of Campania, 80138 Naples, Italy; 5 Department of Cardiology, Medical University of Warsaw, 02-091 Warsaw, Poland; 6 Chair of Cardiology, University of Campania-Monaldi Hospital, 80131 Napoli, Italy

**Keywords:** Emery–Dreifuss muscular dystrophy (EDMD1), X-chromosome inactivation (XCI), cardiac symptoms, skewed X-chromosome inactivation

## Abstract

X-linked Emery–Dreifuss muscular dystrophy (EDMD1) affects approximately 1:100,000 male births. Female carriers are usually asymptomatic but, in some cases, they may present clinical symptoms after age 50 at cardiac level, especially in the form of conduction tissue anomalies. The aim of this study was to evaluate the relation between heart involvement in symptomatic EDMD1 carriers and the X-chromosome inactivation (XCI) pattern. The XCI pattern was determined on the lymphocytes of 30 symptomatic and asymptomatic EDMD1 female carriers—25 familial and 5 sporadic cases—seeking genetic advice using the androgen receptor (AR) methylation-based assay. Carriers were subdivided according to whether they were above or below 50 years of age. A variance analysis was performed to compare the XCI pattern between symptomatic and asymptomatic carriers. The results show that 20% of EDMD1 carriers had cardiac symptoms, and that 50% of these were ≥50 years of age. The XCI pattern was similar in both symptomatic and asymptomatic carriers. Conclusions: Arrhythmias in EDMD1 carriers poorly correlate on lymphocytes to a skewed XCI, probably due to (a) the different embryological origin of cardiac conduction tissue compared to lymphocytes or (b) the preferential loss of atrial cells replaced by fibrous tissue.

## 1. Introduction 

X-linked Emery–Dreifuss muscular dystrophy (OMIM 310300; EDMD1) affects about 1:100,000 males. It is caused by mutations in the *EDMD1* gene [1,2], which encodes the emerin protein—a component of the inner nuclear membrane in the muscles (skeletal, smooth, and cardiac muscle) and other tissues including skin and blood (leukocytes) [3,4,5]. The phenotype is characterized by triad joint contractures (elbows, Achilles tendons, and posterior cervical muscles), humeroperoneal muscle weakness, and cardiac involvement as conduction disturbances [6,7,8,9,10,11,12]. Although female carriers of EDMD1 are usually asymptomatic, they can sometimes present clinical symptoms such as cardiac arrhythmias, including atrial fibrillation or atrioventricular (AV) block [3,13,14,15,16,17,18]. These can lead to sudden cardiac death [19,20]. There are no data available on the prevalence of symptoms in female EDMD1 carriers, although cardiac symptoms seem to correlate with age [13]. On the contrary, no peripheral myopathy and contractures have been reported [19]. It has been suggested that cardiac symptoms in EDMD1 carriers depend on the deficiency of the emerin protein in the nuclei of cells [3]. Previous studies have demonstrated decreased levels of emerin in muscles, skin, leukocytes, and lymphoblastoid cell lines [3,4]. However, a reduction of around 50% in protein was not associated with symptoms [4], while reductions of >95% were observed in symptomatic carriers [3]. It has also been suggested that the amount of protein reduction may depend on skewed X-chromosome inactivation (XCI) [3,14]. However, only one study has reported the analysis of XCI in EDMD1 carriers, and only one was symptomatic among them [3].

We reported the results of XCI analysis in EDMD1 carriers to study the potential role of skewed XCI in the pathogenesis of cardiac symptoms. In particular, we tested 30 EDMD1 carriers and analyzed the results observed in symptomatic compared to asymptomatic subjects. 

## 2. Subjects and Methods 

### 2.1. Subjects 

Thirty EDMD1 carriers, 25 from 9 families and 5 females related to sporadic cases, were included in the study. The diagnosis of the EDMD1 carrier was based on the family history and confirmed by molecular analysis in familial and sporadic cases. Mutations in the *EMD* gene are shown in Table 1.

The carriers were divided into two groups: symptomatic (n = 5) and asymptomatic (n = 25). Furthermore, the last group was subdivided by <50 or ≥50 years of age, as arrhythmic events usually occur in EDMD1 carriers after the age of 50. The carriers were classified as symptomatic or asymptomatic according to the presence or absence of clinical symptoms, respectively. Muscle strength was assessed by manual muscle testing using the Medical Research Council (MRC) scale [21]. Heart involvement was investigated through a complete cardiological examination including standard electrocardiogram (ECG), 24 h Holter monitoring, and echocardiography. A prolonged PQ interval (the time from the onset of the P wave to the start of the QRS complex), a decreased amplitude of P wave, an increased dispersion of P wave, and the presence of atrial fibrillation/flutter, atrial or ventricular ectopic beats, or AV blocks of variable degrees, observed at the electrocardiogram; a sinus pause of >2.5 s, the presence of atrial fibrillation/flutter, atrial or ventricular ectopic beats, or AV blocks of variable degrees observed at the ECG Holter monitoring; increased atrial or ventricular dimensions, a reduced ejection fraction (EF; and wall motion abnormalities observed at the echocardiography were considered pathological.

On the contrary, we defined EDMD1 carriers who did not show any sign of muscle impairment, contractures, and/or cardiomyopathy as asymptomatic. 

### 2.2. Methods 

#### 2.2.1. DNA Analysis 

The peripheral blood was collected into tubes containing ethylenediaminetetraacetic acid (EDTA). Genomic DNA was extracted from leukocytes using the standard operating procedures (SOPs) established by the EuroBioBank Network and quantified spectrophotometrically. An amount of 1 μL was electrophoresed in 1% agarose gel to evaluate DNA integrity. 

DNA samples were provided by the Naples Human Mutation Gene Biobank (NHMGB) genetic biobank of Cardiomyology and Medical Genetics, which is a member of EuroBioBank and the Telethon Network of Genetic Biobanks (TNGB). 

At least one EDMD1-carrying male in each family was analyzed in order to differentiate between the mutant (XCm) and wild-type (XCw) alleles. All subjects, and/or a legal representative in the case of a minor, provided written informed consent to the study for blood collection, in accordance with the Declaration of Helsinki. The study was approved by the Ethical Committee of the University of Campania “Luigi Vanvitelli” (00923-16). 

#### 2.2.2. X-Inactivation Assay 

The pattern of X-chromosome inactivation was determined as previously reported [22,23]. In particular, 500 ng of genomic DNA samples was digested with 10 units *Hpa*II and 10 units *Hha*II methylation-sensitive enzymes in 50 μL of sterile distilled water at 37 °C overnight. Then, 500 ng from each of the 88 DNA samples were incubated overnight with the same volumes of buffer and distilled water as above but excluding enzymes as the control. Digested and undigested DNA samples were used as templates for the amplification of the androgen receptor (AR) in exon 1. The PCR was performed in a 25 μL reaction volume composed of 2 μL digested or undigested DNA, PCR buffer (10 mM Tris-HCl; 50 mM KCl; pH 8.3), 5 pmol forward primer marked with the WellRED dye-labeled D4-PA at 5’ (Sigma Aldrich, Milan, Italy), 5 pmol reverse primer, 1.5 mM MgCl_2_, 0.5 mM dNTP, and 0.3 U GoTaq DNA polymerase (Promega, Milan, Italy). The PCR conditions were as follows: 95 °C for 5 min, 28 × (95 °C for 30 s, 62 °C for 30 s, 72 °C for 30 min), and 72 °C for 7 min. 

The sequences of the oligonucleotides used were as follows:

- Forward 5’-[D4-PA]TCCAGAATCTGTTCCAGAGCGTGC-3’;
- Reverse 5’-ATGAGGAACAGCAACCTTCACAGC-3’.

**Figure 1:**
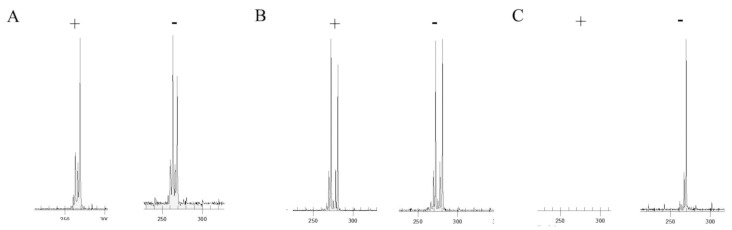
Gene scanner traces for *Hpa*II digested (+) and undigested (−) DNA. The peak represents the amplified androgen receptor (AR) allele. The size of the allele is determined by the number of repeats within the *AR* gene. The area under the peak indicates the degree of amplification of the alleles. The higher peaks correspond to the expected size of alleles, while the shorter peaks should be considered as artifacts. One carrier (**A**) presented a skewed XCI (20:80), while the other showed a random X-chromosome invitation (XCI) (**B**). The digested DNA sample of the Emery–Dreifuss muscular dystrophy (EDMD1) male (**C**) did not show a peak (negative control), while the undigested sample presented one peak.

To separate the two alleles, 1 µL of PCR product was mixed with 38.5 µL of Sample Loading Solution (Beckman, Fullerton, CA, USA) and 0.5 μL of DNA Size Standard Kit 400. This standard included DNA fragments labeled with WellRED fluorescent dye of the following sizes: 60, 70, 80, 90, 100, 120, 140, 160, 180, 190, 200, 220, 240, 260, 280, 300, 320, 340, 360, 380, 400, and 420 nucleotides (Beckman, Fullerton, CA, USA). The mix was loaded on a 96-well plate and 1 drop of mineral oil was added to each well. The fragment analysis was performed by means of a Beckman CEQ 8000 Genetic Analysis System, using the Frag-3 run method. PCR products derived from the undigested DNA samples that gave 1 peak, similar to the CAG repeats, were considered uninformative for the analysis. The ratio between the two alleles (peak areas of allele 1 digested/undigested)/[(allele 1 peak digested/undigested) + (allele 2 digested/undigested)] × 100 was used to analyze the distribution of XCI degree in our cohort of carriers. The higher peaks corresponding to the expected size of alleles were considered for the analysis, while the shorter peaks were considered polymerase artifacts (such as stutter and plus A peaks). The degree of XCw inactivation in the digested DNA in carriers with positive family history was calculated as follows: peak area of (XCw digested/undigested)/[(XCw digested/undigested) + (XCm digested/undigested)] × 100. The allele 2 inactivation was calculated as follows: peak area of the (allele 2 digested/undigested)/[(allele 2 digested/undigested) + (allele 1 digested/undigested)] × 100, considering the shorter as allele as allele 1 and the longer allele as allele 2. The degree of XCI was defined as random when it showed values ≤60%, skewed when the value was between 60% and 85%, and extremely skewed when it was >85% [24,25] (Figure 1). 

### 2.3. Statistical Analysis 

Fisher’s exact test was used to compare the frequency of skewed XCI between carriers <50 or ≥50 years of age. Between symptomatic and asymptomatic carriers, the values were expressed as mean ± SEM. Significance was recognized when *p* < 0.05.

## 3. Results

### 3.1. Subjects

**Figure 2:**
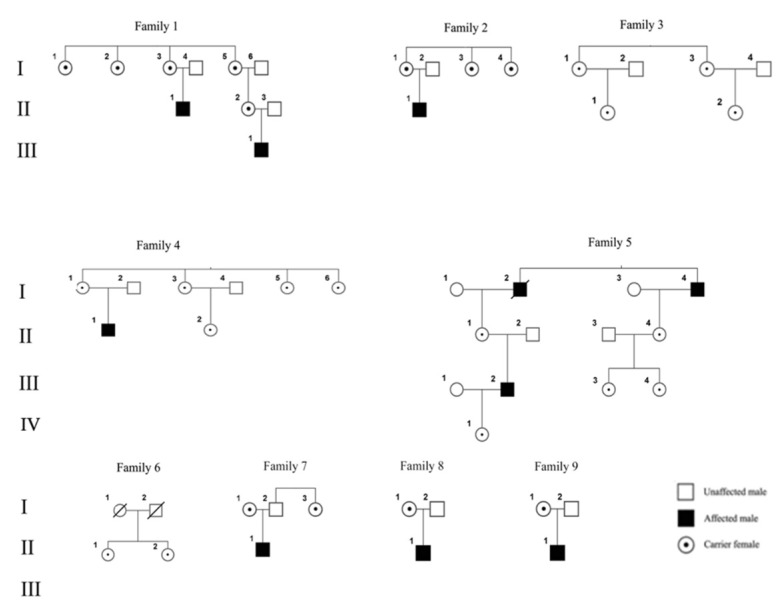
Pedigree of EDMD1 families. Please note that only carriers included in the study are shown.

Twenty five out of 30 EDMD1 carriers analyzed were from nine families (Figure 2) with a positive history for EDMD1, and at least one male affected available. Five carriers were sporadic subjects further included in the analysis, for which it was impossible to differentiate XCw. Five families were from Italy (Sardinia and South Italy) and four were from Poland. Out of the 30 carriers, 10 were ≥50 years of age. The clinical data of the subjects, mutations in the *EMD* gene, and the results of the XCI for familial and sporadic cases are shown in Table 1. 

The average age of familial carriers was 39.1 ± 2.5 years. The average age of sporadic cases, all mothers of affected males, was 47.2 ± 2.2 years. The average age of symptomatic carriers of both familial and isolated cases was 54.6 ± 2.3 years, compared to 37.6 ± 2.2 years of those without symptoms. No carrier <50 years of age was symptomatic. Out of 10 carriers ≥50 years of age, 50% presented cardiac involvement in the form of an AV block of first or second degree ( n = 4), or atrial fibrillation ( n = 1). The carrier with ID 9637 in the isolated case’s group was implanted with a pacemaker due to a second degree of AV block.

Twelve mutations in the *EMD* gene were found in our cohort. Of these, nine are already reported in the UMD-EMD database (http://www.umd.be/EMD/). Two novel mutations were reported by our research team in previous papers [26,27], while one new mutation, c. 564–565 del CT, has not previously been published. 

### 3.2. Analysis of XCI 

**Figure 3:**
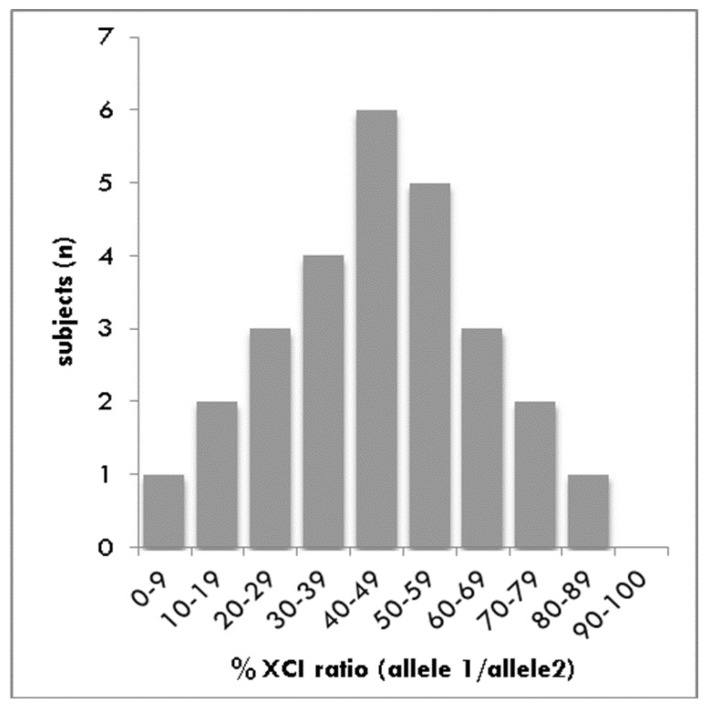
Distribution of XCI ratios in EDMD1 carriers. Normal distribution of the XCI pattern in the leukocytes from the analyzed EDMD1 carriers.

Two carriers were not included in the analysis because they were uninformative. The X-inactivation pattern calculated as the ratio between allele 1 and allele 2 followed a normal distribution with a peak at 40–49 (Figure 3) in EDMD1 carriers. The frequency of skewed or extremely skewed XCI in our cohort of carriers was 35.7%, or 10 out of 28. In particular, 31.5% of carriers were <50 years of age, while 44.4% were ≥50 years of age. 

### 3.3. Statistical Differences 

No significant differences in the frequency of skewed XCI between EDMD1 carriers <50 or ≥50 years of age were found (*p* = 0.06), nor between asymptomatic versus symptomatic carriers (*p* = 0.2). 

## 4. Discussion 

EDMD1 carriers usually do not present symptoms. Very few studies can be found in related literature reporting anecdotal cases of symptomatic carriers, in particular, at the cardiac level [19]. Heart involvement in both affected males and carriers is characterized by the occurrence of conduction defects, such as tachyarrhythmias (ectopic beats, atrial flutter, and fibrillation) or bradyarrhythmias, such as sinoatrial or atrioventricular blocks, often requiring a pacemaker or defibrillator implantation. A higher prevalence of cardiac alterations can be observed in EDMD1 carriers ≥50 years of age.

In this study, the reported data confirm that EDMD1 carriers do not present muscle symptoms but often develop cardiac conduction anomalies. In our group, cardiac abnormalities were observed in approximately 20% of cases when considered as a whole. However, focusing on the ages of the carriers, a higher prevalence (50%) of cardiac disturbances—atrial fibrillation in one carrier, and an AV block of various degrees in four carriers—was observed in carriers ≥50 years. This percentage is well above the figures of arrhythmias in the general population, in which a prevalence of about 2–3% for atrial fibrillation and 6–7% for atrioventricular blocks has been reported in the white population ≥60 years of age [28,29,30,31]. 

Unaffected females in the general population show a normal distribution of the XCI pattern and skewed XCI increasing with age [32], with a prevalence of about 20–40% in females ≥50 years of age [32,33]. Moreover, a skewed XCI, with the preferential inactivation of the XCw, seems to correlate with the development of clinical symptoms in female carriers of X-linked diseases, such as hemophilia B [34], dyskeratosis congenita [35], and Duchenne and Becker muscular dystrophies [36,37]. Some authors suggest that a skewed XCI could play a role in the EDMD1 carrier’s clinical presentation [3,15]. Manilal et al. [3,4] demonstrated reduced levels of emerin from a Western bloting in lymphocytes, lymphoblastoid cell lines (LCL), muscles, and, by immunofluorescence, a mosaic pattern of emerin expression in skin biopsy [3,5]. However, in that study, the XCI analysis was performed on only one subject [3]. This study showed that cardiac symptoms in EDMD1 carriers are unrelated to a skewed XCI on lymphocytes. We found 10 out of 28 (35.7%) cases of skewed or extremely skewed XCI with a growing trend with increasing age, in agreement with previous studies [32,33]. However, no significant correlation was found between age and skewed XCI pattern nor between symptomatic and asymptomatic EDMD1 carriers in our cohort. 

To explain this apparent discordance between heart involvement and degree of the XCI in EDMD1 carriers, besides the already mentioned “age effect”, the different embryological origin of cardiac conduction tissue compared with myocardium should be taken into account. In fact, cardiomyocytes originate as blood cells from the mesoderm germinal layer [38] and share a similar XCI pattern [39,40]. The conduction system consists of highly specialized cardiomyocytes, is innervated by cardiac ganglia, and presents a high number of fibroblasts. The latter derive from two different germinal layers (ectodermal and mesodermal) [38,41,42,43] exhibiting a different XCI pattern [39,40]. As cardiac symptoms in EDMD1 are prevalently supraventricular arrhythmias or atrioventricular blocks that involve the conduction tissue, we cannot exclude that the XCI pattern analyzed in leukocytes does not reflect that of the conduction tissue, unlike what we observed and reported in Duchenne and Becker carriers who present myocardial failure [23]. Furthermore, at least two more factors may play a role in the pathogenesis of cardiac disturbances in EDMD1 and explain our results: (1) the marked loss of atrial cells replaced by fibrous tissue [43,44,45,46], and (2) the intrinsic alterations of the conduction system where emerin is localized, in particular at the intercalated disks [47]. However, future studies on animal models are necessary to confirm our hypothesis. 

A limitation of our study may be the small number of subjects analyzed to confirm these data and establish definite results. Another could be the failure to analyze the XCI pattern in cardiac conduction tissue obtained by endomyocardial biopsy, which was not performed due to ethical reasons. However, this paper represents the first analysis of XCI in a group of genetically confirmed EDMD1 female carriers.

## Figures and Tables

**Table 1:** Clinical and genetic data, and X-chromosome inactivation (XCI) ratios in Emery–Dreifuss muscular dystrophy (EDMD1) carriers, are analyzed. XCw: X-chromosome wild-type; AV: atrioventricular.

Family Number	ID Subject	Age at Last Control (Years)	XCw Inactivation (%)	Signs/Symptoms	*EMD* Mutation
**N. 1**					
**I-1**	5327	**58**	54.0	1^st^ degree AV block	c. 130 C>T
**I-2**	5328	**51**	49	no	
**I-4**	5325	49	30	no	
**I-3**	5326	47	70	no	
**II-2**	5322	28	28	no	
**N. 2**					
**I-1**	4811	32	35.4	no	c. 564–565 del CT*
**I-2**	4810	24	40.7	no	
**I-3**	4809	21	49.7	no	
**N. 3**					
**I-1**	9265	**50**	64.8	no	c. 1A>G
**I-3**	9266	46	41.9	no	
**II-1**	9264	31	65.5	no	
**II-2**	9267	25	50.9	no	
**N. 4**					
**I-1**	9577	**52**	91.8	1^st^ degree AV block	c. IVS4+2G>Cc. 399+2G>C
**II-2**	9261	35	44.8	no	
**I-5**	9262	22	34.3	no	
**I-6**	9263	20	0	no	
**N. 5**					
**IV-1**	9259	29	n.i.	no	c. 153delC
**III-3**	9258	**53**	89.2	no	
**III-4**	9257	49	50.	no	
**N. 6**					
**II-1**	9269	37	40.7	no	c. 451dup
**II-2**	9270	25	68.9	no	
**N. 7**					
**I-1**	6418	42	90.8	no	c. 106 A>T **
**I-2**	5008	40	70	no	
**N. 8**					
**I-1**	2907	**50**	35.3	1^st^ degree AV block	c. 740 C<T***
**N. 9**					
**I-1**	202	**62**	20	Atrial fibrillation	c. 740 C<T***
**Isolated Cases**	**ID Subject**	**Age at Last Control (Years)**	**XCw Inactivation (%)**	**Cardiac Findings**	***EMD*** **Mutation**
**1**	8581	**50**	n.i.	no	c. 106 A>T*
**2**	9268	40	48.9	no	c.192_192delinsTC
**3**	9256	44	78.8	no	c.IVS3 −27del18
**4**	9637	**51**	74.6	2^nd^ degree AV block	c.IVS2+1G>A
**5**	9578	**51**	18.7	no	c.256 C>T
